# Prognosis prediction of α-FAtE score for locoregional immunotherapy in hepatocellular carcinoma

**DOI:** 10.3389/fimmu.2024.1496095

**Published:** 2025-01-10

**Authors:** Zehao Zheng, Renguo Guan, Rongce Zhao, Junyu Gan, Xinhao Xiong, Jing-wen Zou, Shaohua Li, Qiaoxuan Wang, Wei Wei, Jie Mei, Rongping Guo

**Affiliations:** ^1^ Department of Liver Surgery, Sun Yat-sen University Cancer Center, Guangzhou, Guangdong, China; ^2^ State Key Laboratory of Oncology in South China, Collaborative Innovation Center for Cancer Medicine, Guangzhou, Guangdong, China; ^3^ Guangdong Provincial Clinical Research Center for Cancer, Guangzhou, Guangdong, China; ^4^ Department of General Surgery, Guangdong Provincial People’s Hospital (Guangdong Academy of Medical Sciences), Southern Medical University, Guangzhou, Guangdong, China; ^5^ Department of Dermatologic Surgery and Dermatologic Oncology, Dermatology Hospital of Southern Medical University, Guangdong Provincial Dermatology Hospital, Guangzhou, Guangdong, China

**Keywords:** hepatocellular carcinoma, locoregional therapy, immunotherapy, alpha-fetoprotein, alkaline phosphatase, eosinophil

## Abstract

**Purpose:**

The α-FAtE score, composed of alpha-fetoprotein, alkaline phosphatase, and eosinophil levels, has been reported as a predictor of prognosis in hepatocellular carcinoma (HCC) patients treated with atezolizumab plus bevacizumab. This study aimed to investigate the predictive ability of α-FAtE score for the efficacy and safety of locoregional immunotherapy as the treatment of HCC patients.

**Methods and patients:**

We conducted a retrospective study of 446 HCC patients at Sun Yat-sen University Cancer Center from January 1^st^ 2019 to January 1^st^ 2023. The predictive performance was evaluated by the concordance index, the area under the receiver operating characteristics curve, the Kaplan-Meier curve and multiple Cox regression analysis.

**Results:**

446 patients were divided into the α-FAtE 0-1 group (n=211) and α-FAtE 2-3 group (n=235). The median progression-free survival(PFS) of the α-FAtE 0-1 group and 2-3 group was 7.3 months (95%CI 6.6-8.7 months), and 12.3 months (95% CI 10.4–14.1 months; *P*<0.001), respectively. The median overall survival (OS) of the α-FAtE 0-1 group and 2-3 group was 16.3 months (95%CI 13.7-21.5 months) and 34.1 months (95% CI 27.6–NA months; *P*<0.001), respectively. HCC patients in the α-FAtE 2-3 group had higher complete response (CR) rate and experienced less drug-related adverse events than those in the α-FAtE 0-1 group. Moreover, a lower α-FAtE score was identified as an independent prognostic indicator for both OS and PFS of advanced HCC patients receiving locoregional immunotherapy.

**Conclusion:**

The α-FAtE score is a superior predictor of prognosis in HCC patients receiving locoregional immunotherapy, offering a valuable tool for patient stratification and treatment planning.

## Introduction

Hepatocellular carcinoma (HCC), one of the typical fatal cancers worldwide, accounts for nearly 80%-90% of primary liver tumors (1). The majority of HCC patients are diagnosed in an advanced, even terminal stage, resulting in poor prognosis (2). Recent advances in the locoregional and systemic management of advanced HCC have brought the promise of improved HCC prognosis closer to reality (3, 4). Transarterial therapies, including transarterial chemoembolization (TACE) and hepatic arterial infusion chemotherapy (HAIC), can effectively control the local tumor by delivering chemotherapeutic drugs directly to the liver tumor sites through the hepatic artery to increase anti-tumor effect (5, 6). Simultaneously, atezolizumab plus bevacizumab (A+T) and durvalumab plus tremelimumab are superior to sorafenib in prolonging overall survival in HCC patients (7, 8). However, the objective response rate (ORR) of monotherapy in patients with advanced HCC remains unsatisfactory. Recent studies have suggested that the combination of locoregional therapy and immunotherapy has synergistic effects and may provide a better clinical benefit for advanced-staged HCCs (9–11).

A variety of biomarkers may be able to identify HCC patients who could benefit from locoregional immunotherapy, including Neutrophil-to-Lymphocyte ratio (12), CRAFITY score (13, 14), CCL28, and Betacellulin (9). So far, most biomarkers have not been validated in large and multi-center populations. The α-FAtE score, which is based on alpha-fetoprotein (AFP), alkaline phosphatase (ALP), and eosinophil, is a new predictive score that could predict the response and prognosis of HCC patients who treated with atezolizumab plus bevacizumab (15). However, the predictive role of the α-FAtE score for locoregional immunotherapy remains unknown and warrants further validation. Therefore, this present study retrospectively included 446 HCC patients receiving locoregional immunotherapy, to evaluate and validate the predictive ability of the α-FAtE score for the determination of the prognosis.

## Materials and methods

### Study design and participants

From January 1^st^ 2019 to January 1^st^ 2023, 648 HCC patients confirmed with advanced HCC and treated with locoregional immunotherapy at Sun Yat-sen University Cancer Center were candidates for study enrollment. The inclusion criteria are: 1) diagnosed by imaging examinations or biopsy pathology based on American Association for the Study of Liver Diseases (AASLD) guidelines (16); 2) aged between 18 to 80 years; 3) had treated with at least one locoregional therapy and 2 cycles immunotherapy; 4) had a performance status (PS) score ≤ 2; 5) liver functions at Child-Pugh A or B stage; 6) had no other malignancies; and 7) had complete follow-up data and laboratory data of Eosinophil, AFP and ALP. Exclusion criteria as follows: 1) <18 or >80 years; 2) liver function reserve at Child-Pugh C; 3) had other malignancies history; 4) incomplete Eosinophil, AFP and ALP data or follow-up data; 5) patients who died within 30 days after receiving locoregional-immunotherapy. This retrospective study was approved by the ethics committee of Sun Yat-sen University Cancer Center (B2020-190-01) and was performed in full accordance with the Declaration of Helsinki and the STROCSS criteria (17). The data were collected, reviewed, de-identified, and anonymized prior to analysis, and the ethics committee waived the requirement for informed consent.

### Treatment of locoregional-immunotherapy

Locoregional therapy included TACE and HAIC. The operation procedure of locoregional therapy was described in previous studies (18, 19). The category and dosages of immunotherapy agents applied in these patients are provided in **Supplementary Table S1**. ICI therapy was administered intravenously after locoregional therapy. Advanced HCC patients received locoregional immunotherapy every 3 to 4 weeks until disease progression, unacceptable toxicity, treatment abandonment, or death. All patients underwent regular clinical evaluations, laboratory blood tests, contrast-enhanced computed tomography (CT), or magnetic resonance imaging (MRI) examination to evaluate the initial tumor situation and treatment response according to the modified RECIST (mRECIST) (20). The definition (13) and cutoff value of other serum biomarkers were based on previous studies (21–25).

### Data collection and study outcomes

The laboratory blood test data (including Eosinophil, AFP, and ALP), demographic information, and tumor characteristics used in this study were retrospectively collected from the medical record system within 2 weeks before the initial locoregional therapy. According to a previous study, the α-FAtE score consisted of three blood indicators levels at baseline, and the score of each indicator was defined as follows: AFP <400 ng/mL =1 point and ≥400 ng/mL =0 point; ALP ≤125 IU/L =1 point and >125IU/L=0 point; eosinophil count ≥70/μL=1 point and <70/μL=0 point (15). The α-FAtE score of each patient was calculated based on the sum of individual indicator scores. OS and PFS were the primary outcomes, while tumor response and treatment safety were the second outcomes. Severity of drug-related AEs were assessed based on the Common Terminology Criteria for Adverse Events version 5.0 of National Cancer Institute.

### Statistical analysis

Cox proportional hazards regression was utilized to calculate the hazard ratio (HR) and 95%CI and evaluate the prognostic value for each variable. R package *SurvCorr* was used to calculate the relations [ρ(95% CI)] between OS and PFS (26, 27). The concordance index (C-index) and the area under the receiver operating characteristics curve (ROC curve) were utilized to evaluate the discrimination performance of α-FAtE score and other inflammation biomarkers. Similar to a previous study (28), inverse probability of treatment-weighting (IPTW) and propensity score matching (PSM) methods were both used to minimize the differences between the α-FAtE 0-1 group and the α-FAtE 2-3 group. Survival analysis was performed using the Kaplan-Meier curve analysis and comparisons of survival rates were estimated using the Log-rank test. α-FAtE 0-1 score group and α-FAtE 2-3 score group were compared by using the independent t-test for continuous data and the χ2 test for categorical data. All the statistical analyses were performed by R version 4.3.1 software (http://www.r-project.org/) and SPSS 20.0. A two-tailed *P*-value<0.05 was regarded as statistically significant in all tests.

## Results

### Patient characteristics

**Figure 1:**
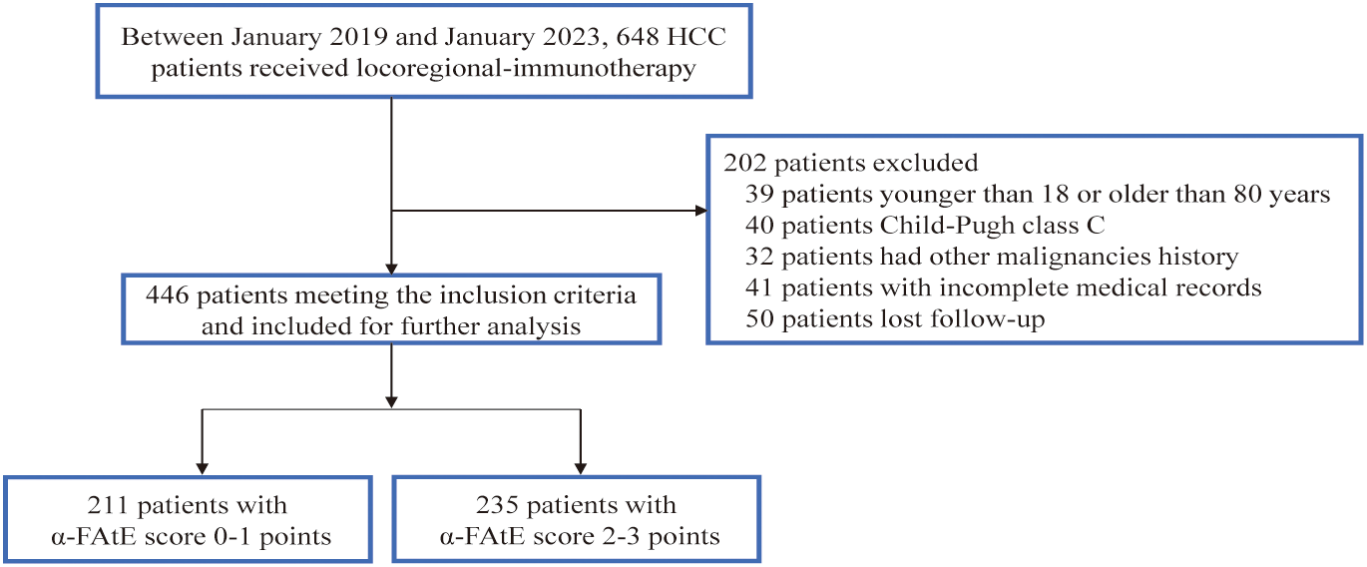
Flow chart of the study.

From January 1^st^ 2019 to January 1^st^ 2023, a total of 446 advanced HCC patients who received locoregional immunotherapy met the criteria and were eventually included in the present study (**Figure 1**). **Table 1** showed the demographic and clinical characteristics of these enrolled patients. Among the 446 patients, 390 (87.44%) were male and 104 (23.32%) were older than 60 years. A total of 386 (86.55%) patients had positive hepatitis B surface antigen, with most patients at Barcelona Clinic Liver Cancer stage C and had good liver function reserve. There were 389 patients treated with anti-PD1 and 57 with anti-PDL1 inhibitors. HAIC, TACE, and HAIC plus TACE were conducted as locoregional treatments in 349 (78.25%), 8 (1.79%), and 89 (19.96%) patients, respectively. Moreover, 371 patients received tyrosine kinase inhibitor (TKI) combination treatments. 211 HCC patients were allocated to the group with α-FAtE 0-1 (**Supplementary Figure S1**), and 52.69% (n = 235) were allocated to the α-FAtE 2-3 group(**Supplementary Figure S2**). Further information on the relationship between different α-FAtE group and clinicopathological characteristics were provided in **Table 1**.

**Table 1:** Baseline clinicopathological characteristics of HCC patients.

Patient Characteristics	Total (N=446)	α-FAtE 2–3 point group (n= 235)	α-FAtE 0–1 point group (n= 211)	*P*-value
Age				0.02
<60	342 (76.68%)	169 (71.91%)	173 (81.99%)	
≥60	104 (23.32%)	66 (28.09%)	38 (18.01%)	
Gender				1
male	390 (87.44%)	20687.66%)	184 (87.20%)	
female	56 (12.56%)	29 (12.34%)	27 (12.8%)	
Immunotherapy				1
PD1	386 (86.55%)	203 (86.38%)	183 (86.73%)	
PDL1	60 (13.45%)	32 (13.62%)	28 (13.27%)	
Targeted therapy				0.02
without	82 (18.39%)	53 (22.55%)	29 (13.74%)	
with	364 (81.61%)	182 (77.45%)	182 (86.26%)	
Locoregional therapy				0.39
HAIC	349 (78.25%)	178 (75.74%)	171 (81.04%)	
TACE	8 (1.79%)	5 (2.13%)	3 (1.42%)	
HAIC plus TACE	89 (19.96%)	52 (22.13%)	37 (17.54%)	
Tumor number				0.36
single	107 (23.99%)	61 (25.96%)	46 (21.80%)	
multiple	339 (76.01%)	174 (74.04%)	165 (78.2%)	
Tumor size				<0.01
<5cm	38 (8.52%)	35 (14.89%)	3 (1.42%)	
≥5cm and <10cm	157 (35.20%)	96 (40.85%)	61 (28.91%)	
≥10cm	251 (56.28%)	104 (44.26%)	147 (69.67%)	
Macrovascular invasion				<0.01
without	158 (35.43%)	108 (45.96%)	50 (23.7%)	
with	288 (64.57%)	127 (54.04%)	161 (76.3%)	
Extrahepatic metastasis				0.02
without	205 (45.96%)	121 (51.49%)	84 (39.81%)	
with	241 (54.04%)	114 (48.51%)	127 (60.19%)	
BCLC				<0.01
A	31 (6.95%)	23 (9.79%)	8 (3.79%)	
B	64 (14.35%)	43 (18.3%)	21 (9.95%)	
C	351 (78.70%)	169 (71.91%)	182 (86.26%)	
HBsAg				<0.01
negative	60 (13.45%)	42 (17.87%)	18 (8.53%)	
positive	386 (86.55%)	193 (82.13%)	193 (91.47%)	
ALBI				<0.01
1	272 (60.99%)	159 (67.66%)	113 (53.55%)	
2-3	174 (39.01%)	76 (32.34%)	98 (46.45%)	
Child-Pugh				
Class A	400 (89.69%)	218 (92.77%)	182 (86.26%)	
Class B	46 (10.31%)	17 (7.23%)	29 (13.74%)	
AST	85.39 ± 92.98	63.18 ± 61.41	110.12 ± 113.81	
ALT	55.21 ± 47.87	50.79 ± 43.79	60.15 ± 51.71	
ALB	41.25 ± 4.83	41.85 ± 4.92	40.59 ± 4.64	
TBIL	19.15 ± 20.37	17.30 ± 23.10	21.20 ± 16.64	
WBC	7.41 ± 2.67	7.14 ± 2.52	7.70 ± 2.81	
Neutrophil	5.59 ± 6.29	5.10 ± 5.33	6.14 ± 7.17	
lymphocyte	1.65 ± 1.37	1.79 ± 1.79	1.51 ± 0.60	
Hemoglobin	143.10 ± 22.23	141.01 ± 19.74	145.43 ± 24.55	
Platelet	266.45 ± 124.22	Mean ± SD	231.64 ± 96.75	
CRP	29.44 ± 38.41	23.60 ± 36.54	35.95 ± 39.48	
AFP				<0.01
<400	141 (31.61%)	127 (54.04%)	14 (6.64%)	
≥400	291 (65.25%)	105 (45.96%)	186 (93.36%)	
CRAFITY				<0.01
1	67 (15.02%)	62 (26.38%)	5 (2.37%)	
2	192 (43.05%)	121 (51.49%)	71 (33.65%)	
3	187 (41.93%)	52 (22.13%)	135 (63.98%)	
PNI				0.03
0	105 (23.54%)	45 (10.09%)	60 (13.45%)	
1	341 (76.46%)	190 (42.60%)	151 (33.86%)	
NLR				<0.01
0	360 (80.72%)	205 (45.96%)	155 (34.75%)	
1	86 (19.28%)	30 (6.73%)	56 (12.56%)	
PLR				<0.01
0	216 (48.43%)	130 (29.15%)	86 (19.28%)	
1	230 (51.57%)	105 (23.54%)	125 (28.03%)	
SII				<0.01
0	66 (14.80%)	49 (10.99%)	17 (3.81%)	
1	380 (85.20%)	186 (41.70%)	194 (43.50%)	
LCR				<0.01
0	376 (84.30%)	179 (40.13%)	197 (44.17%)	
1	70 (15.70%)	56 (12.56%)	14 (3.14%)	
CAR				<0.01
0	262 (58.74%)	159 (35.65%)	103 (23.09%)	
1	184 (41.26%)	76 (17.04%)	108 (24.22%)	
mGPS				<0.01
0	120 (26.91%)	88 (19.73%)	32 (7.17%)	
1	290 (65.02%)	131 (29.37%)	159 (35.65%)	
2	36 (8.07%)	16 (3.59%)	20 (4.48%)	

α-FAtE, α-fetoprotein (AF), alkaline phosphatase (A) and eosinophil count (E); BCLC, Barcelona Clinic Liver Cancer; HBsAg, hepatitis B surface antigen; AST, aspartate aminotransferase; ALT, alanine aminotransferase; ALB, albumin; TBil, total bilirubin; WBC, leukocyte; CRP, C-reaction protein; AFP, alpha-fetoprotein; CRAFITY, C-reactive protein and alpha-fetoprotein in immunotherapy; PNI, prognostic nutritional index; NLR, neutrophil-to-lymphocyte ratio; PLR, platelet-to-lymphocyte ratio; SII, systemic immune-inflammation index; LCR, lymphocyte-to-CRP ratio; CAR, CRP-to-albumin ratio; mGPS, modified Glasgow prognostic score.

### Prognosis of patients

The median follow-up duration was 28.5 months (95%CI 27.3-32.2 months), while the median OS time was 24.6 months (95%CI 20.4-30.4 months). 218 patients died and 324 patients experienced tumor progression during the follow‐up period.

**Figure 2:**
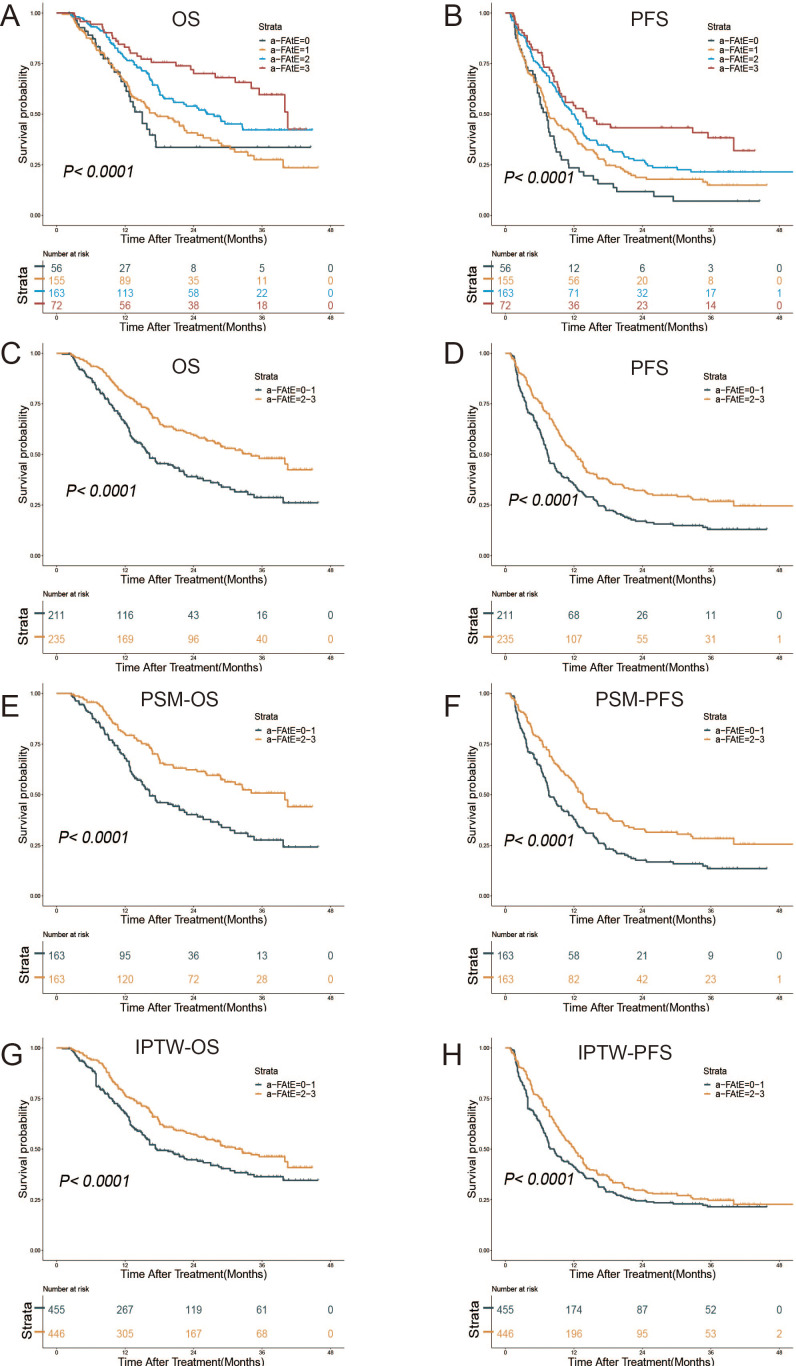
Kaplan–Meier curves for overall survival (OS) and progression-free survival (PFS) of patients after locoregional immunotherapy. **(A, B)** Survival outcomes in the α-FAtE score 0 to 3 groups, **(A)** OS, **(B)** PFS. **(C–H)** Survival outcomes in the α-FAtE 0-1 group and 2-3 group, **(C)** OS, **(D)** PFS; **(E)** OS after PSM, **(F)** PFS after PSM; **(G)**OS after IPTW, **(H)** and PFS after IPTW **(H)**. α-FAtE, α-fetoprotein (AF), alkaline phosphatase (A), and eosinophil count (E); PSM, propensity score matching; IPTW, inverse probability of treatment-weighting.

Based on the initial definition from Rossari et al. (15), the HCC patients were firstly divided into four groups. The results showed that decreased levels of α-FAtE score had poorer OS (*P*<0.001; **Figure 2A**) and PFS (*P*<0.001; **Figure 2B**). Similar to Rossari. et.al., we further grouped patients into α-FAtE 0–1 group and 2–3 group and conducted the Kaplan–Meier analysis of the OS and PFS time. The median OS of the α-FAtE 0-1 group and 2-3 group was 16.3 months (95%CI 13.7-21.5 months) and 34.1 months (95% CI 27.6–NA months; *P*<0.0001; **Figure 2C**), respectively. The median PFS of the α-FAtE 0-1 group and 2-3 group was 7.3 months (95%CI 6.6-8.7 months), 12.3 months (95% CI 10.4–14.1 months; *P*<0.0001; **Figure 2D**), respectively. To further minimize the differences between α-FAtE 0-1 score group and α-FAtE 2-3 score group, IPTW and PSM were both conducted. Interestingly, the α-FAtE 2-3 score group still had a longer OS and PFS compared to the α-FAtE 0-1 score group after adjustment for PSM (**Figures 2E, F**) and IPTW (**Figures 2G, H**). Therefore, the α-FAtE score could be a useful predictor for the prognosis of HCC patients with locoregional immunotherapy.

We further investigated the prognostic value of the α-FAtE score in different patients (with TKI treatment vs. without TKI treatment). As the **Supplementary Figures S3A, B** showed, we found the α-FAtE precisely stratified the prognosis of HCC patients treated with TKI (both *P*< 0.05). We also found the similar curve in predicting OS for HCC patients without Targeted drug treatment(*P*< 0.05). However, the median PFS of the α-FAtE 0-1 group and 2-3 group in the HCC patients without TKI treatment was 9.8 months (95%CI 6.6-NA months) and 40 months (95%CI 8.7-NA months), respectively (*P*= 0.13; **Supplementary Figure S3B**).

### Tumor response and α-FAtE score

We performed the radiological assessment for tumor treatment response using mRECIST criteria (**Table 2**). 35 (7.85%) and 210 (47.09%) HCC patients achieved CR and partial response(PR), respectively, while 130 (29.15%) and 64 (14.35%) experienced stable disease(SD) and progressive disease (PD), respectively. The tumor overall response rate (ORR) and the disease control rate (DCR) of the whole cohort were 55.61%, and 84.53%, respectively. The CR rate of patients in the α-FAtE 2-3 group was significantly higher than those in the α-FAtE 0-1 group (6.05% vs.1.79%, *P*<0.05). The ORR of the α-FAtE 2-3 group and 0-1 group was 58.72%, and 52.13%, respectively (*P*=0.21). The DCR- of the α-FAtE 0-1 score and 2-3 score was 80.57%, and 87.23%, respectively (*P*= 0.15).

**Table 2:** The relationship between α-FAtE score and tumor response.

Characteristics	α-FAtE 2–3 point group (n= 235)	α-FAtE 0–1 point group (n= 211)	Total (N=446)	*P*-value
CR	27 (6.05%)	8 (1.79%)	35 (7.85%)	6.7e-3
SD	67 (28.51%)	62 (29.38%)	129 (28.92%)	0.92
PD	27 (6.05%)	37 (8.30%)	64 (14.35%)	0.16
PR	111 (24.89%)	99 (22.20%)	210 (47.09%)	0.96
ORR	138 (58.72%)	110 (52.13%)	248 (55.61%)	0.21
DCR	205 (87.23%)	172 (80.57%)	377 (84.53%)	0.15

CR, complete response; PR, partial response; SD, stable disease; PD, progression disease; ORR, objective response rate; DCR, disease control rate. α-FAtE, α-fetoprotein (AF), alkaline phosphatase (A), and eosinophil count (E).

### Safety profile

**Table 3** provided the overall incidence of drug-related adverse events. No adverse-related death was found during drug administration. The adverse events of any grade were observed in 164(69.79%) patients in the α-FAtE 2-3 group and 168(79.62%) in the α-FAtE 0-1 group (*P*=0.02). Grade 3 or 4 adverse events were observed in 29 (12.34%) of α-FAtE 2-3 group and 37(17.54%) of the α-FAtE 0-1 group (*P*=0.14). Patients in the α-FAtE 0-1 group were more likely to have a fever, liver injury, or digestive hemorrhage than those within the α-FAtE 2-3 score group.

**Table 3:** Drug-related adverse events according to the α-FAtE score.

	α-FAtE 0–1point group (n= 211)	α-FAtE 2–3point group (n= 235)	*P*-value
AEs of any grade	168	164	0.02
AEs of Grade≥3	37	29	0.14
Fever			
any grade	30	15	<0.01
Grade≥3	1	0	0.96
Abdominal pain			
Any grade	71	59	0.06
Grade≥3	6	10	0.59
Diarrhea			
Any grade	23	13	0.06
Grade≥3	1	0	0.96
Cough			
Any grade	17	22	0.75
Grade≥3	1	0	0.96
Vomiting			
Any grade	19	15	0.39
Grade≥3	1	0	0.96
Skin rash			
Any grade	25	14	0.16
Grade≥3	4	4	1
Insomnia			
Any grade	7	6	0.84
Grade≥3	0	0	1
Liver injury			
Any grade	110	99	0.04
Grade≥3	23	15	0.12
Hypothyroidism			
Any grade	3	3	1
Grade≥3	0	1	0.96
Hypertension			
Any grade	12	8	0.35
Grade≥3	0	0	1
Digestive hemorrhage			
Any grade	6	0	0.03
Grade≥3	3	0	0.21

α-FAtE, α-fetoprotein (AF), alkaline phosphatase (A), and eosinophil count (E); AEs, Adverse events.

### Correlation between the OS and PFS by the α-FAtE score

The coefficient (ρ value) was subsequently calculated in order to estimate the correlation between OS and PFS. For all patients in this study, PFS had the strong correlation with OS (ρ = 0.67, 95% CI 0.60-0.73). Furthermore, the subgroup analysis by the α-FAtE score revealed that both the α-FAtE 0-1 group (ρ = 0.65, 95% CI 0.54-0.73) and the α-FAtE 2-3 group (ρ = 0.69, 95% CI 0.58-0.78) had a high correlation with OS.

### Independent factors associated with PFS and OS

Univariate Cox regression analysis was used to estimate the potential risk factors of PFS and OS among α-FAtE score and clinicopathological factors (**Supplementary Table S2**). Schoenfeld residual analysis showed that extrahepatic metastasis and LCR did not meet the proportional hazards assumption test for OS, and immunotherapy, targeted therapy, and BCLC stage did not meet the proportional hazards assumption test for PFS (**Supplementary Table S3**). These clinical variables were all regarded as stratified factors during the analysis. Finally, α-FAtE score was regarded as an independent prognostic factor for both OS (HR = 0.71; 95% CI 0.52-0.97; *P*= 0.03) and PFS (HR = 0.74; 95% CI 0.58-0.96; *P*= 0.02) (**Table 4**).

**Table 4:** Multivariate Cox regression for progression-free survival and overall survival.

Multivariate Cox regression analysis						
Variables	Progression-free Survival			Overall Survival		
	HR	95%CI	*P*-value	HR	95%CI	*P*-value
α-FAtE (0-1/2-3)	0.74	0.58-0.96	**0.02**	0.71	0.52-0.97	**0.03**
Immunotherapy (PD1/PDL1)				0.50	0.31-0.82	**0.005**
Targeted therapy (without/with)				1.34	0.90-2.00	0.15
Tumor number (single/multiple)	1.42	1.03-1.97	**0.03**	1.50	1.06-2.12	**0.02**
Macrovascular invasion (without/with)				1.10	0.82-1.49	0.52
Extrahepatic metastasis (without/with)	1.15	0.84-1.56	0.38			
ALBI (1/2-3)	1.19	0.88-1.62	0.25	1.49	1.02-2.16	**0.038**
CRAFITY						
0	Reference					
1	0.93	0.62-1.39	0.72	1.01	0.61-1.68	0.97
2	1.06	0.66-1.71	0.81	1.21	0.67-2.19	0.54
NLR	0.97	0.72-1.32	0.87	1.16	0.81-1.66	0.42
PLR	1.11	0.86-1.43	0.42	1.04	0.76-1.42	0.80
PNI (<45/≥45)	0.95	0.67-1.35	0.79	1.10	0.72-1.67	0.65
SII (0/1)	1.44	0.97-2.14	0.07	1.64	0.99-2.71	0.051
CAR (0/1)	1.01	0.74-1.36	0.97	0.92	0.64-1.32	0.64
mGPS						
0	Reference					
1	0.91	0.60-1.37	0.72	1.14	0.68-1.89	0.62
2	1.05	0.58-1.88	0.81	1.55	0.78-3.09	0.21

α-FAtE, α-fetoprotein (AF), alkaline phosphatase (A) and eosinophil count (E); CRAFITY, C-reactive protein and alpha-fetoprotein in immunotherapy; PNI, prognostic nutritional index; NLR, neutrophil-to-lymphocyte ratio; PLR, platelet-to-lymphocyte ratio; SII, systemic immune-inflammation index; CAR, CRP-to-albumin ratio; mGPS, modified Glasgow prognostic score.

Bold values means P-value<0.05.

### The prediction performance of other serum biomarkers and the α-FAtE score

**Figure 3:**
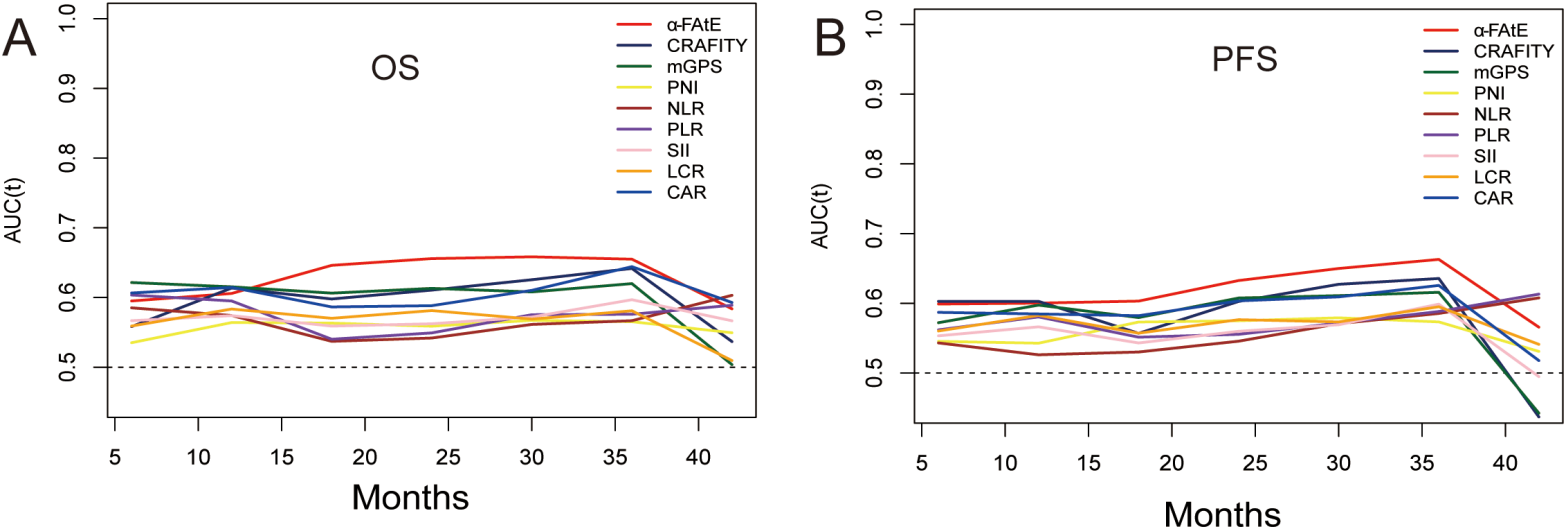
Time-dependent ROC cures of the α-FAtE score and other blood biomarkers for overall survival **(A)** and progression-free survival **(B)**. α-FAtE, α-fetoprotein (AF), alkaline phosphatase (A) and eosinophil count (E); CRAFITY, C-reactive protein and alpha-fetoprotein in immunotherapy; PNI, prognostic nutritional index; NLR, neutrophil-to-lymphocyte ratio; PLR, platelet-to-lymphocyte ratio; SII, systemic immune-inflammation index; LCR, lymphocyte-to-CRP ratio; CAR, CRP-to-albumin ratio; mGPS, modified Glasgow prognostic score.

To further compare the prognostic prediction performance of the α-FAtE score and common serum biomarkers, the ROC curves were calculated. The results indicated α-FAtE had a better capacity to estimate OS of HCC patients receiving locoregional immunotherapy than other blood biomarkers (**Figure 3A**). In term of PFS, the α-FAtE score was similar to other blood biomarkers in the prediction of PFS at 12 months, but was better at 24 and 36 months (**Figure 3B**). The c-index of the α-FAtE score was 0.596 for OS and 0.575 for PFS. As summarized in the **Supplementary Table S4**, the ROC curves of the α-FAtE for PFS at 12-, 24-, and 36- months were 0.60, 0.633, and 0.663, respectively, and for OS at 12-, 24-, and 36- months were 0.606, 0.656, and 0.647, respectively.

## Discussion

Identifying and validating the predictive and prognostic biomarkers is a major challenge in the management of advanced-staged HCC. Currently, there are no well-validated biomarkers to identify the HCC patients who could benefit from a combination of locoregional therapy and immunotherapy. Genomics characteristics could provide large-scale screening of specific prognostic biomarkers for advanced HCC treated with locoregional therapy (29) and immunotherapy (30, 31). However, the necessity for an invasive biopsy, in addition to the high financial cost, limits its clinical application. Radiomics models using the imaging parameters and machine learning methods are non-invasive and have outstanding prediction performance (32, 33). Nonetheless, reproducibility, generalizability and model interpretability are major obstacles of these clinically applied radiomics (34, 35). Serum biomarkers including inflammatory biomarkers (36), liver functional biomarkers and tumor biomarkers (37, 38) are reported as useful biomarkers for advanced HCC patients. Each individual serum biomarker is easily and widely accessible but lack good sensitivity and specificity. Thus, we believed that a satisfactory biological indicator must be non-invasive, simple and cost-effective. Moreover, it must also be precise and possess good generalizability and interpretability. Rossari et al. (15) provided a novel and simple composite α-FAtE score, which is composed of baseline AFP, ALP, and eosinophils levels. Their multicenter retrospective study revealed that α-FAtE had excellent prediction ability for the tumor therapeutic response and prognosis of advanced HCC patients treated with atezolizumab plus bevacizumab. In this current study, we evaluated the predictive ability of the α-FAtE score among 446 advanced HCC patients with locoregional immunotherapy. According to the α-FAtE score, 446 patients were further divided into 0-1 group(n=211) and 2-3 group(n=235). We found that the α-FAtE score could effectively and accurately stratify the survival time of patients. HCC patients in the α-FAtE 2-3 group had higher CR rates than those in the α-FAtE 0-1 group. Many patients in the α-FAtE 0-1 group experienced more drug-related adverse events. Furthermore, the α-FAtE score was further identified as an independent risk factor for both OS and PFS. Thus, the α-FAtE score had the great predictive value for the tumor response and prognosis in advanced HCC receiving combination of locoregional therapy and immunotherapy.

The reason that the α-FAtE score had the prognostic value in advanced HCC patients receiving combination of locoregional therapy and immunotherapy is possibly due to it represented the tumor characteristics, reflected the liver function reserve and was associated with tumor microenvironments. Serum AFP levels are abnormally elevated in approximately 70% of HCC patients (39, 40). Numerous studies have suggested that serum AFP levels could serve as a predictive indicator for treatment evaluation in early, intermediate, and advanced stage HCC (41–44). Consistent with other studies, we found that patients with AFP >400ng/ml had a worse prognosis. AFP expression and secretion of HCC were positively associated with tumor characteristics (tumor burden, aggressiveness, worse tumor differentiation) and negatively correlated with treatment response. The underlying mechanism may be that AFP could promote the malignant cells proliferation and metastasis (45), inhibit the apoptosis induced by chemoagents (46) and facilitate the evasion of the immune surveillance (47). The liver functional reserve plays an important role in therapeutic effects for advanced HCC. ALP is a blood biomarker for the evaluation of liver function. The elevation of ALP indicated liver function damage and a worse prognosis for HCC patients who received anti-PD1 therapy (48) and locoregional therapy (49). The administration of HAIC and TACE as multi-course treatments has been demonstrated to achieve excellent tumour control and prolong survival by delivering the chemotherapy agents to the local tumors. However, it is important to note that high local concentrations of chemotherapy agents may disrupt the balance of damage repair in liver cells, thereby resulting in hepatic impairment (50) and ALP elevation. In addition, the elevated expression of ALP in HCC cells could promote tumor glycolysis (51) and enhance the HCC cell proliferation in the preclinical models. Meanwhile, eosinophil is a prognostic biomarker for multiple tumors, and elevated eosinophil levels are associated with better survival (52–54). HAIC could alter the multicellular ecosystem in HCC and promote the expansion and accumulation of antitumor CD8 T cell (55). Eosinophils can also promote the production of memory CD8 T cells (56) and guide CD8 T cells into the tumor and enhance the anti-tumor effect (57). Moreover, the Eosinophils in the tumor microenvironment can cooperate with CD4 T cells to enhance the treatment response to immune checkpoint inhibitors (58). Therefore, the α-FAtE score had the prognostic values for advanced HCC patients receiving locoregional immunotherapy.

The current research had some limitations as well. First of all, some potential biases in the enrollment of HCC patients due to its retrospective nature might exist. Second, different types of ICI agents were used in our study based on patient preference, household income, and insurance coverage. But on the other hand, this cohort may be more representative of the HCC population in the real world. Finally, 86.55% of the enrolled patients had HBV infection. Subgroup analysis of the IMbrave 150 and HIMALAYA studies both showed that the treatment efficacy of immune checkpoint inhibitors was higher in HBV-related HCC compared to other etiological factors. Therefore, the superior prognostic value of α-FAtE score for patients caused by alcohol, NASH, etc. was still unclear. A large-scale multicenter, and prospective clinical trials are necessary to reconfirm our findings.

## Conclusion

Our study evaluated and validated the superior prognostic value of the α-FAtE score in advanced HCC patients receiving locoregional immunotherapy.

## Data Availability

The raw data supporting the conclusions of this article will be made available by the authors, without undue reservation.

## Supplementary material

Supplementary Figure 1 The radiological imaging of patients in α-FAtE 0-1 group. A 68-year-old man at BCLC B stage (AFP 45542 ng/mL, ALP 157.3 U/L and eosinophils 0.06*109/L) in α-FAtE 0-1 group achieved complete regression after receiving locoregional Immunotherapy. **(A-C)** Before treatments; **(D-F)** After treatments.

Supplementary Figure 2 The radiological imaging of patients in α-FAtE 2-3 group. A 44-year-old man at BCLC C stage (AFP 13.12ng/mL, ALP 77.4U/L and eosinophils 0.05*109/L) in α-FAtE 2-3 group achieved complete regression after receiving locoregional Immunotherapy. **(A-C)** Before treatments; **(D-F)** After treatments.

Supplementary Figure 3 The Kaplan–Meier curves for overall survival **(A)** and progression-free survival **(B)** based on the α-FAtE score for with Targeted therapy group and without Targeted therapy group. α-FAtE: α-fetoprotein (AF), alkaline phosphatase (A) and eosinophil count(E).

## References

[1] Huang DQ Singal AG Kanwal F Lampertico P Buti M Sirlin CB. Hepatocellular carcinoma surveillance - utilization, barriers and the impact of changing aetiology. Nat Rev Gastroenterol Hepatol. (2023) 20:797–809. doi: 10.1038/s41575-023-00818-8 37537332

[2] Singal AG Kanwal F Llovet JM. Global trends in hepatocellular carcinoma epidemiology: implications for screening, prevention and therapy. Nat Rev Clin Oncol. (2023) 20:864–84. doi: 10.1038/s41571-023-00825-3 37884736

[3] Yang C Zhang H Zhang L Zhu AX Bernards R Qin W. Evolving therapeutic landscape of advanced hepatocellular carcinoma. Nat Rev Gastroenterol Hepatol. (2023) 20:203–22. doi: 10.1038/s41575-022-00704-9 36369487

[4] Yang JD Hainaut P Gores GJ Amadou A Plymoth A Roberts LR. A global view of hepatocellular carcinoma: trends, risk, prevention and management. Nat Rev Gastroenterol Hepatol. (2019) 16:589–604. doi: 10.1038/s41575-019-0186-y 31439937 PMC6813818

[5] Zhao M Guo Z Zou YH Li X Yan ZP Chen MS. Arterial chemotherapy for hepatocellular carcinoma in China: consensus recommendations. Hepatol Int. (2024) 18:4–31. doi: 10.1007/s12072-023-10599-6 37864725

[6] Raoul JL Forner A Bolondi L Cheung TT Kloeckner R de Baere T. Updated use of TACE for hepatocellular carcinoma treatment: How and when to use it based on clinical evidence. Cancer Treat Rev. (2019) 72:28–36. doi: 10.1016/j.ctrv.2018.11.002 30447470

[7] Finn RS Qin S Ikeda M Galle PR Ducreux M Kim TY. Atezolizumab plus bevacizumab in unresectable hepatocellular carcinoma. N Engl J Med. (2020) 382:1894–905. doi: 10.1056/NEJMoa1915745 32402160

[8] Sangro B Chan SL Kelley RK Lau G Kudo M Sukeepaisarnjaroen W. Four-year overall survival update from the phase III HIMALAYA study of tremelimumab plus durvalumab in unresectable hepatocellular carcinoma. Ann Oncol. (2024) 35:448–57. doi: 10.1016/j.annonc.2024.02.005 38382875

[9] Lai Z He M Bu X Xu Y Huang Y Wen D. Lenvatinib, toripalimab plus hepatic arterial infusion chemotherapy in patients with high-risk advanced hepatocellular carcinoma: A biomolecular exploratory, phase II trial. Eur J Cancer. (2022) 174:68–77. doi: 10.1016/j.ejca.2022.07.005 35981413

[10] Rimassa L Finn RS Sangro B. Combination immunotherapy for hepatocellular carcinoma. J Hepatol. (2023) 79:506–15. doi: 10.1016/j.jhep.2023.03.003 36933770

[11] Zhang TQ Geng ZJ Zuo MX Li JB Huang JH Huang ZL. Camrelizumab (a PD-1 inhibitor) plus apatinib (an VEGFR-2 inhibitor) and hepatic artery infusion chemotherapy for hepatocellular carcinoma in Barcelona Clinic Liver Cancer stage C (TRIPLET): a phase II study. Signal Transduct Target Ther. (2023) 8:413. doi: 10.1038/s41392-023-01663-6 37884523 PMC10603153

[12] Qu S Wu D Hu Z. Neutrophil-to-lymphocyte ratio and early tumor shrinkage as predictive biomarkers in unresectable hepatocellular carcinoma patients treated with lenvatinib, PD-1 inhibitors, in combination with TACE. Technol Cancer Res Treat. (2023) 22:15330338231206704. doi: 10.1177/15330338231206704 37849287 PMC10585992

[13] Guan R Mei J Lin W Deng M Li S Guo R. Is the CRAFITY score a superior predictor of prognosis and adverse events in hepatocellular carcinoma patients treated with locoregional-immunotherapy? Hepatol Int. (2023) 17:1279–88. doi: 10.1007/s12072-023-10535-8 37129721

[14] Hu ZX Xu XY Wang Z Huang JT Li WC Zhang S. Prognosis prediction of CRAFITY score in HCC undergoing TACE combined with PD-(L)1 inhibitors and molecular targeted therapy. J Hepatocell Carcinoma. (2023) 10:2073–82. doi: 10.2147/JHC.S439660 PMC10676090 38022730

[15] Rossari F Tada T Suda G Shimose S Kudo M Yoo C. alpha-FAtE: A new predictive score of response to atezolizumab plus bevacizumab for unresectable hepatocellular carcinoma. Int J Cancer. (2024) 154:1043–56. doi: 10.1002/ijc.v154.6 37994647

[16] Singal AG Llovet JM Yarchoan M Mehta N Heimbach JK Dawson LA. AASLD Practice Guidance on prevention, diagnosis, and treatment of hepatocellular carcinoma. Hepatology. (2023) 78:1922–65. doi: 10.1097/HEP.0000000000000466 PMC10663390 37199193

[17] Mathew G Agha R Albrecht J Goel P Mukherjee I Pai P. STROCSS 2021: Strengthening the reporting of cohort, cross-sectional and case-control studies in surgery. Int J Surg. (2021) 96:106165. doi: 10.1016/j.ijsu.2021.106165 34774726

[18] Li QJ He MK Chen HW Fang WQ Zhou YM Xu L. Hepatic arterial infusion of oxaliplatin, fluorouracil, and leucovorin versus transarterial chemoembolization for large hepatocellular carcinoma: A randomized phase III trial. J Clin Oncol. (2022) 40:150–60. doi: 10.1200/JCO.21.00608 34648352

[19] Li S Mei J Wang Q Shi F Liu H Zhao M. Transarterial infusion chemotherapy with FOLFOX for advanced hepatocellular carcinoma: a multi-center propensity score matched analysis of real-world practice. Hepatobiliary Surg Nutr. (2021) 10:631–45. doi: 10.21037/hbsn.2020.03.14 PMC8527433 34760967

[20] Lencioni R Llovet JM. Modified RECIST (mRECIST) assessment for hepatocellular carcinoma. Semin Liver Dis. (2010) 30:52–60. doi: 10.1055/s-0030-1247132 20175033 PMC12268942

[21] Proctor MJ Morrison DS Talwar D Balmer SM O’Reilly DS Foulis AK. An inflammation-based prognostic score (mGPS) predicts cancer survival independent of tumour site: a Glasgow Inflammation Outcome Study. Br J Cancer. (2011) 104:726–34. doi: 10.1038/sj.bjc.6606087 PMC3049591 21266974

[22] Grenader T Waddell T Peckitt C Oates J Starling N Cunningham D. Prognostic value of neutrophil-to-lymphocyte ratio in advanced oesophago-gastric cancer: exploratory analysis of the REAL-2 trial. Ann Oncol. (2016) 27:687–92. doi: 10.1093/annonc/mdw012 26787231

[23] Ishizuka M Nagata H Takagi K Iwasaki Y Shibuya N Kubota K. Clinical significance of the C-reactive protein to albumin ratio for survival after surgery for colorectal cancer. Ann Surg Oncol. (2016) 23:900–7. doi: 10.1245/s10434-015-4948-7 26530445

[24] Hu B Yang XR Xu Y Sun YF Sun C Guo W. Systemic immune-inflammation index predicts prognosis of patients after curative resection for hepatocellular carcinoma. Clin Cancer Res. (2014) 20:6212–22. doi: 10.1158/1078-0432.CCR-14-0442 25271081

[25] Okugawa Y Toiyama Y Yamamoto A Shigemori T Ide S Kitajima T. Lymphocyte-C-reactive protein ratio as promising new marker for predicting surgical and oncological outcomes in colorectal cancer. Ann Surg. (2020) 272:342–51. doi: 10.1097/SLA.0000000000003239 32675548

[26] Carpenter DJ Leng J Arshad M Giles W Kirkpatrick JP Floyd SR. Intracranial and extracranial progression and their correlation with overall survival after stereotactic radiosurgery in a multi-institutional cohort with brain metastases. JAMA Netw Open. (2023) 6:e2310117. doi: 10.1001/jamanetworkopen.2023.10117 37099292 PMC10134007

[27] Schemper M Kaider A Wakounig S Heinze G. Estimating the correlation of bivariate failure times under censoring. Stat Med. (2013) 32:4781–90. doi: 10.1002/sim.v32.27 23775542

[28] Guan R Zhang N Deng M Lin Y Huang G Fu Y. Patients with hepatocellular carcinoma extrahepatic metastases can benefit from hepatic arterial infusion chemotherapy combined with lenvatinib plus programmed Death-1 inhibitors. Int J Surg. (2024) 110:4062–73. doi: 10.1097/JS9.0000000000001378 PMC11254277 38549220

[29] Lyu N Wang X Li JB Lai JF Chen QF Li SL. Arterial chemotherapy of oxaliplatin plus fluorouracil versus sorafenib in advanced hepatocellular carcinoma: A biomolecular exploratory, randomized, phase III trial (FOHAIC-1). J Clin Oncol. (2022) 40:468–80. doi: 10.1200/JCO.21.01963 34905388

[30] Spahn S Roessler D Pompilia R Gabernet G Gladstone BP Horger M. Clinical and genetic tumor characteristics of responding and non-responding patients to PD-1 inhibition in hepatocellular carcinoma. Cancers (Basel). (2020) 12:3830. doi: 10.3390/cancers12123830 33353145 PMC7766321

[31] Zhang N Yang X Piao M Xun Z Wang Y Ning C. Biomarkers and prognostic factors of PD-1/PD-L1 inhibitor-based therapy in patients with advanced hepatocellular carcinoma. biomark Res. (2024) 12:26. doi: 10.1186/s40364-023-00535-z 38355603 PMC10865587

[32] Yi X Fu Y Long Q Zhao Y Li S Zhou C. Myosteatosis can predict unfavorable outcomes in advanced hepatocellular carcinoma patients treated with hepatic artery infusion chemotherapy and anti-PD-1 immunotherapy. Front Oncol. (2022) 12:892192. doi: 10.3389/fonc.2022.892192 35651812 PMC9149214

[33] Hua Y Sun Z Xiao Y Li H Ma X Luo X. Pretreatment CT-based machine learning radiomics model predicts response in unresectable hepatocellular carcinoma treated with lenvatinib plus PD-1 inhibitors and interventional therapy. J Immunother Cancer. (2024) 12:e008953. doi: 10.1136/jitc-2024-008953 39029924 PMC11261678

[34] Floca R Bohn J Haux C Wiestler B Zollner FG Reinke A. Radiomics workflow definition & challenges - German priority program 2177 consensus statement on clinically applied radiomics. Insights Imaging. (2024) 15:124. doi: 10.1186/s13244-024-01704-w 38825600 PMC11144687

[35] Wu X You J Zhang S Zhang B. Pretreatment CT-based machine learning radiomics model predicts response in unresectable hepatocellular carcinoma treated with lenvatinib plus PD-1 inhibitors and interventional therapy. J Immunother Cancer. (2024) 12:e010330. doi: 10.1136/jitc-2024-010330 39326887 PMC11425937

[36] Xiao Y Zhu G Xie J Luo L Deng W Lin L. Pretreatment neutrophil-to-lymphocyte ratio as prognostic biomarkers in patients with unresectable hepatocellular carcinoma treated with hepatic arterial infusion chemotherapy combined with lenvatinib and camrelizumab. J Hepatocell Carcinoma. (2023) 10:2049–58. doi: 10.2147/JHC.S432134 PMC10642375 37965074

[37] Xiao Y Chen W Deng W Zhu G Xie J Luo L. Prognostic value of alpha-fetoprotein in unresectable hepatocellular carcinoma treated with hepatic artery infusion chemotherapy combined with lenvatinib and camrelizumab. J Hepatocell Carcinoma. (2024) 11:1251–63. doi: 10.2147/JHC.S460922 PMC11228324 38979083

[38] Zou X Xu Q You R Yin G. Correlation and efficacy of TACE combined with lenvatinib plus PD-1 inhibitor in the treatment of hepatocellular carcinoma with portal vein tumor thrombus based on immunological features. Cancer Med. (2023) 12:11315–33. doi: 10.1002/cam4.v12.10 PMC10242346 36951443

[39] Baig JA Alam JM Mahmood SR Baig M Shaheen R Sultana I. Hepatocellular carcinoma (HCC) and diagnostic significance of A-fetoprotein (AFP). J Ayub Med Coll Abbottabad. (2009) 21:72–5. 20364746

[40] Hu X Chen R Wei Q Xu X. The landscape of alpha fetoprotein in hepatocellular carcinoma: where are we? Int J Biol Sci. (2022) 18:536–51. doi: 10.7150/ijbs.64537 PMC8741863 35002508

[41] Wang MD Sun LY Qian GJ Li C Gu LH Yao LQ. Prothrombin induced by vitamin K Absence-II versus alpha-fetoprotein in detection of both resectable hepatocellular carcinoma and early recurrence after curative liver resection: A retrospective cohort study. Int J Surg. (2022) 105:106843. doi: 10.1016/j.ijsu.2022.106843 35995351

[42] Wang Y Chen Y Ge N Zhang L Xie X Zhang J. Prognostic significance of alpha-fetoprotein status in the outcome of hepatocellular carcinoma after treatment of transarterial chemoembolization. Ann Surg Oncol. (2012) 19:3540–6. doi: 10.1245/s10434-012-2368-5 22532305

[43] Deng M Lei Q Wang J Lee C Guan R Li S. Nomograms for predicting the recurrence probability and recurrence-free survival in patients with hepatocellular carcinoma after conversion hepatectomy based on hepatic arterial infusion chemotherapy: a multicenter, retrospective study. Int J Surg. (2023) 109:1299–310. doi: 10.1097/JS9.0000000000000376 PMC10389618 37038994

[44] Sun X Mei J Lin W Yang Z Peng W Chen J. Reductions in AFP and PIVKA-II can predict the efficiency of anti-PD-1 immunotherapy in HCC patients. BMC Cancer. (2021) 21:775. doi: 10.1186/s12885-021-08428-w 34218801 PMC8254996

[45] Lu Y Zhu M Li W Lin B Dong X Chen Y. Alpha fetoprotein plays a critical role in promoting metastasis of hepatocellular carcinoma cells. J Cell Mol Med. (2016) 20:549–58. doi: 10.1111/jcmm.2016.20.issue-3 PMC4759472 26756858

[46] Zhu M Li W Lu Y Dong X Chen Y Lin B. Alpha fetoprotein antagonizes apoptosis induced by paclitaxel in hepatoma cells *in vitro* . Sci Rep. (2016) 6:26472. doi: 10.1038/srep26472 27255186 PMC4891737

[47] Zhang M Liu K Zhang Q Xu J Liu J Lin H. Alpha fetoprotein promotes polarization of macrophages towards M2-like phenotype and inhibits macrophages to phagocytize hepatoma cells. Front Immunol. (2023) 14:1081572. doi: 10.3389/fimmu.2023.1081572 36911723 PMC9995430

[48] Zheng Z Mei J Guan R Zhang J Xiong X Gan J. A novel liver-function-indicators-based prognosis signature for patients with hepatocellular carcinoma treated with anti-programmed cell death-1 therapy. Cancer Immunol Immunother. (2024) 73:158. doi: 10.1007/s00262-024-03713-6 38834790 PMC11150358

[49] Guo L Ren H Pu L Zhu X Liu Y Ma X. The prognostic value of inflammation factors in hepatocellular carcinoma patients with hepatic artery interventional treatments: A retrospective study. Cancer Manag Res. (2020) 12:7173–88. doi: 10.2147/CMAR.S257934 PMC7520139 33061563

[50] Mei J Yu C Shi F Guan R Li S Zhong C. The ARH score, a practical guide to decision-making for retreatment with hepatic arterial infusion chemotherapy in hepatocellular carcinoma patients. Int Immunopharmacol. (2024) 138:112551. doi: 10.1016/j.intimp.2024.112551 38950459

[51] al-Rashida M Iqbal J. Inhibition of alkaline phosphatase: an emerging new drug target. Mini Rev Med Chem. (2015) 15:41–51. doi: 10.2174/1389557515666150219113205 25694083

[52] Hude I Sasse S Brockelmann PJ von Tresckow B Momotow J Engert A. Leucocyte and eosinophil counts predict progression-free survival in relapsed or refractory classical Hodgkin Lymphoma patients treated with PD1 inhibition. Br J Haematol. (2018) 181:837–40. doi: 10.1111/bjh.2018.181.issue-6 28439879

[53] Takeuchi E Kondo K Okano Y Ichihara S Kunishige M Kadota N. Pretreatment eosinophil counts as a predictive biomarker in non-small cell lung cancer patients treated with immune checkpoint inhibitors. Thorac Cancer. (2023) 14:3042–50. doi: 10.1111/1759-7714.15100 PMC10599974 37669914

[54] Buder-Bakhaya K Hassel JC. Biomarkers for clinical benefit of immune checkpoint inhibitor treatment-A review from the melanoma perspective and beyond. Front Immunol. (2018) 9:1474. doi: 10.3389/fimmu.2018.01474 30002656 PMC6031714

[55] Huang Y Du Z Lai Z Wen D Huang L He M. Single-nucleus and spatial transcriptome profiling delineates the multicellular ecosystem in hepatocellular carcinoma after hepatic arterial infusion chemotherapy. Adv Sci (Weinh). (2024), e2405749. doi: 10.1002/advs.202405749 39686623 PMC11791974

[56] Zhou J Liu J Wang B Li N Liu J Han Y. Eosinophils promote CD8(+) T cell memory generation to potentiate anti-bacterial immunity. Signal Transduct Target Ther. (2024) 9:43. doi: 10.1038/s41392-024-01752-0 38413575 PMC10899176

[57] Carretero R Sektioglu IM Garbi N Salgado OC Beckhove P Hammerling GJ. Eosinophils orchestrate cancer rejection by normalizing tumor vessels and enhancing infiltration of CD8(+) T cells. Nat Immunol. (2015) 16:609–17. doi: 10.1038/ni.3159 25915731

[58] Blomberg OS Spagnuolo L Garner H Voorwerk L Isaeva OI van Dyk E. IL-5-producing CD4(+) T cells and eosinophils cooperate to enhance response to immune checkpoint blockade in breast cancer. Cancer Cell. (2023) 41:106–23 e10. doi: 10.1016/j.ccell.2022.11.014 36525971

