# Supplementary figures and images for: Prognosis prediction of α-FAtE score for locoregional immunotherapy in hepatocellular carcinoma
Source: Front Immunol. 2025 Jan 10;15:1496095. doi: 10.3389/fimmu.2024.1496095 (PMC11757168; doi:10.3389/fimmu.2024.1496095)

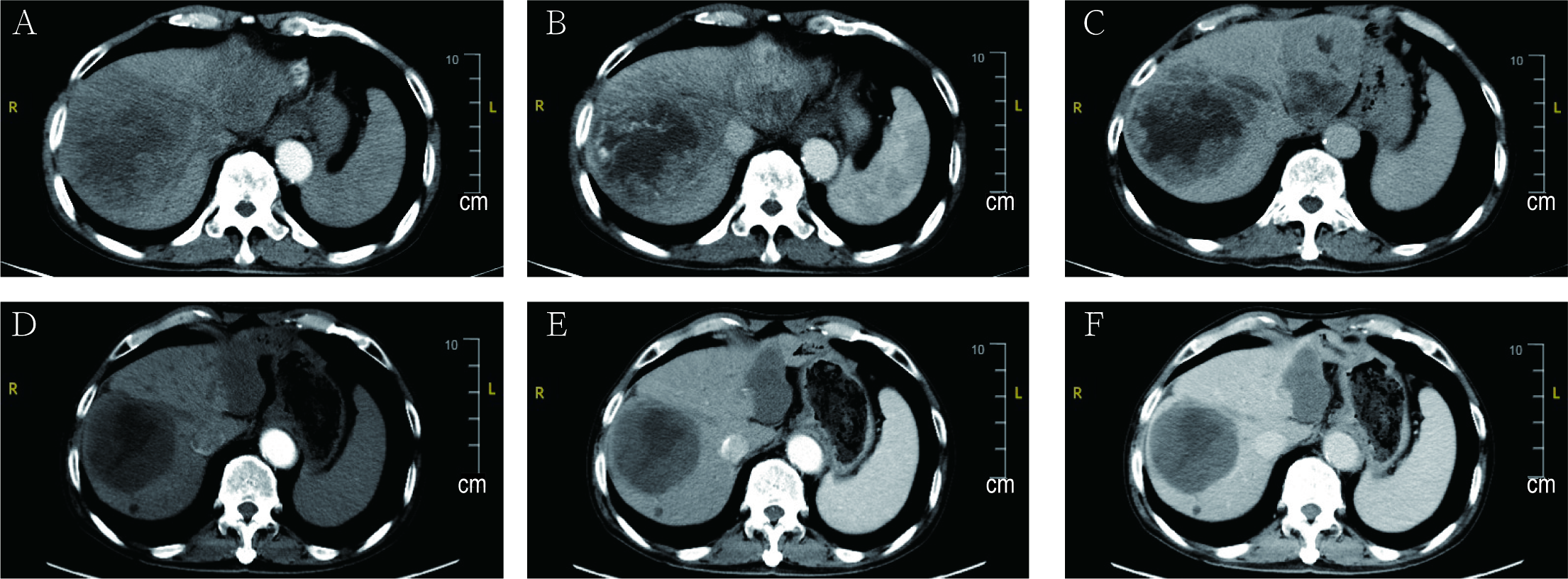

Supplement: Supplementary Figure 1 — The radiological imaging of patients in α-FAtE 0-1 group. A 68-year-old man at BCLC B stage (AFP 45542 ng/mL, ALP 157.3 U/L and eosinophils 0.06*109/L) in α-FAtE 0-1 group achieved complete regression after receiving locoregional Immunotherapy. (A-C) Before treatments; (D-F) After treatments. [file Image1.tif]

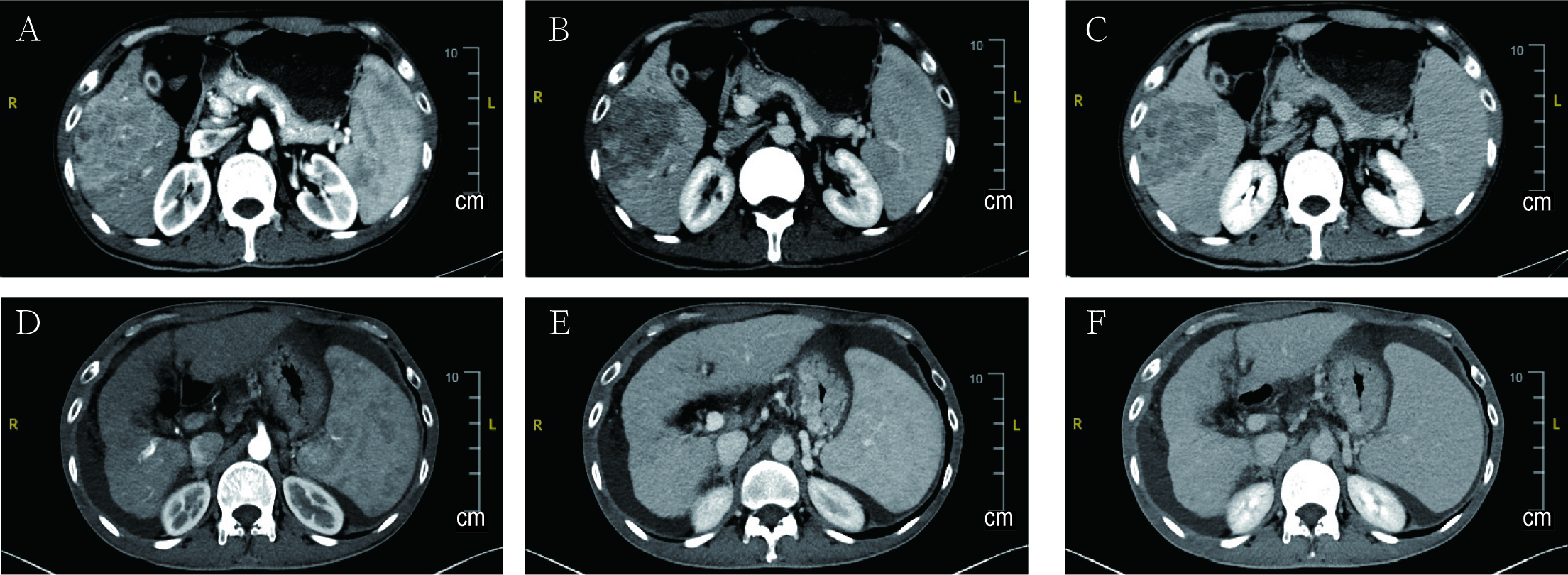

Supplement: Supplementary Figure 2 — The radiological imaging of patients in α-FAtE 2-3 group. A 44-year-old man at BCLC C stage (AFP 13.12ng/mL, ALP 77.4U/L and eosinophils 0.05*109/L) in α-FAtE 2-3 group achieved complete regression after receiving locoregional Immunotherapy. (A-C) Before treatments; (D-F) After treatments. [file Image2.tif]

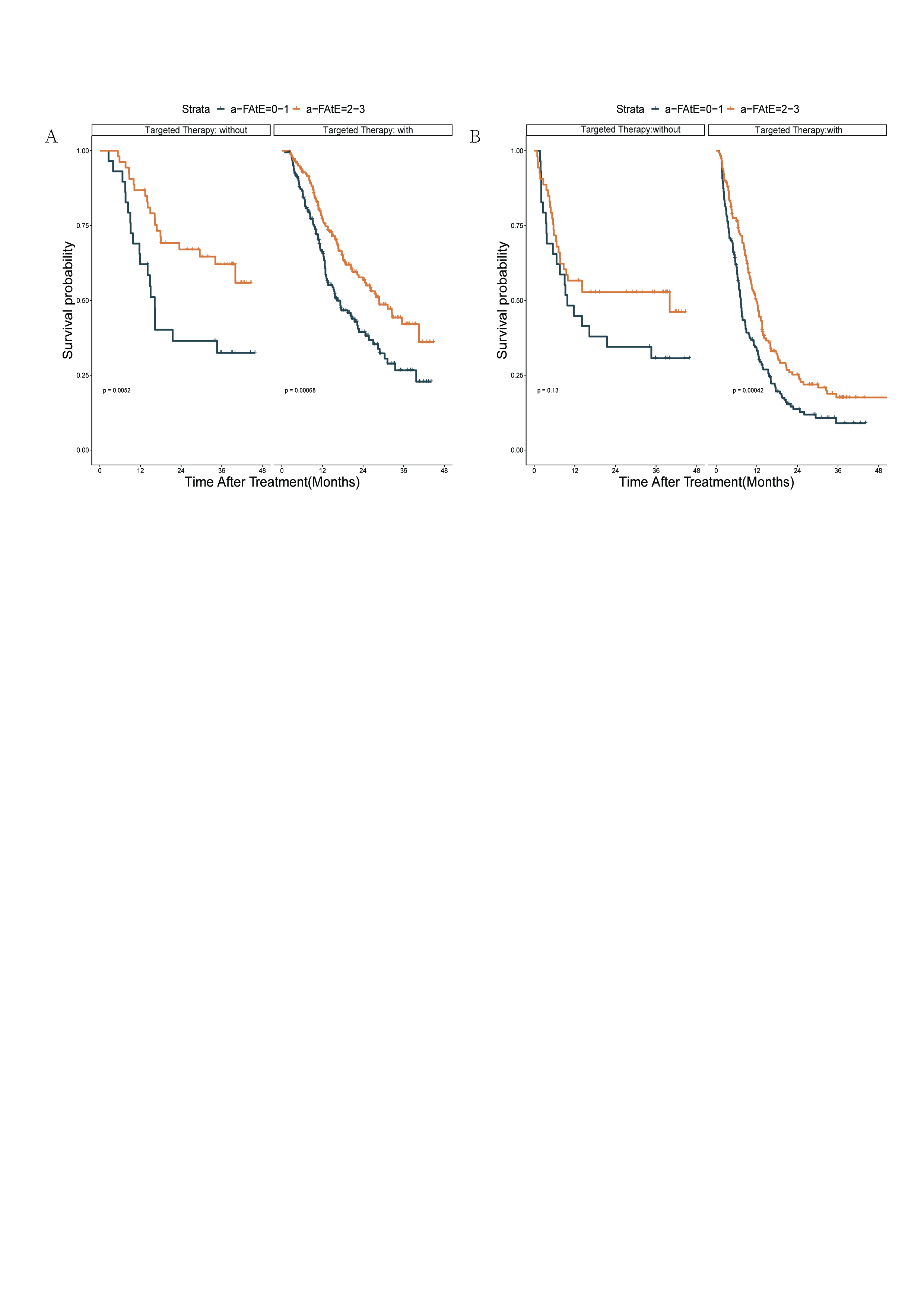

Supplement: Supplementary Figure 3 — The Kaplan–Meier curves for overall survival (A) and progression-free survival (B) based on the α-FAtE score for with Targeted therapy group and without Targeted therapy group. α-FAtE: α-fetoprotein (AF), alkaline phosphatase (A) and eosinophil count(E). [file Image3.tif]
