# Supplementary material for: Prognosis prediction of α-FAtE score for locoregional immunotherapy in hepatocellular carcinoma
Source: Front Immunol. 2025 Jan 10;15:1496095. doi: 10.3389/fimmu.2024.1496095 (PMC11757168; doi:10.3389/fimmu.2024.1496095)
Supplement: Supplementary file 4 [file Table1.docx]

| Supplementary table1: Category and dosage of PD-1 inhibitors | | |
| --- | --- | --- |
| Category | Dose(mg) | No. (%) |
| Durvalumab |  | 58(13.04%) |
| Tislelizumab | 200 | 55(12.33%) |
| Toripalimab | 240 | 142(31.84%) |
| Sintilimab | 200 | 131(29.37%) |
| Pembrolizumab | 200 | 37 (8.3%) |
| Nivolumab | 100 | 6(1.35%) |
| Camrelizumab | 200 | 17(3.81%) |

Abbreviations: No.: Number.

|  | | | | | | | |
| --- | --- | --- | --- | --- | --- | --- | --- |
| Supplementary table 2: Univariate Cox regression for progression-free survival and overall survival | | | | | | | |
| Variables | Progression-free Survival | | | | Overall Survival | | |
|  | HR | 95%CI | *P*-value | | HR | 95%CI | *P*-value |
| α-FAtE(0-1/2-3) | 0.62 | 0.50-0.77 | | <0.001 | 0.55 | 0.42-0.71 | <0.001 |
| Gender(male/female) | 1.14 | 0.82-1.57 | | 0.442 | 1.21 | 0.83-1.78 | 0.319 |
| Age(＜60/≥60) | 0.83 | 0.64-1.08 | | 0.17 | 0.98 | 0.71-1.34 | 0.876 |
| Immunotherapy  (PD1/ PDL1) | 0.65 | 0.46-0.92 | | 0.0154 | 0.53 | 0.34-0.84 | 0.0073 |
| Targeted therapy  (without/with) | 1.89 | 1.38-2.59 | | <0.001 | 1.467 | 1.03-2.08 | 0.0317 |
| Tumor number  (single/multiple) | 1.67 | 1.27-2.2 | | <0.001 | 1.52 | 1.10-2.11 | 0.011 |
| Tumor size |  |  | |  |  |  |  |
| (＜5cm/≥5cm and ＜10cm) | 0.83 | 0.54-1.28 | | 0.412 | 0.99 | 0.57-1.73 | 0.974 |
| (＜5cm/≥10cm) | 1.27 | 1.16-0.25 | | 0.248 | 1.52 | 0.89-2.59 | 2.588 |
| Macrovatbscular  invasion(without/with) | 1.19 | 0.95-1.5 | | 0.131 | 1.37 | 1.03-1.81 | 0.0306 |
| Extrahepatic metastasis (without/with) | 1.63 | 1.3-2.03 | | <0.001 | 1.32 | 1.01-1.73 | 0.0413 |
| BCLC |  |  | |  |  |  |  |
| (A/B) | 1.46 | 0.84-2.54 | | 0.184 | 1.13 | 0.61-2.08 | 0.701 |
| (A/C) | 1.75 | 1.07-2.87 | | 0.0255 | 1.48 | 0.87-2.52 | 0.147 |
| HBsAg(negative/positive) | 1.04 | 0.75-1.44 | | 0.819 | 0.77 | 0.54-1.11 | 0.158 |
| ALBI(1/2-3) | 1.50 | 1.20-1.87 | | <0.001 | 1.73 | 1.32-2.26 | <0.001 |
| Child-Pugh(A/B) | 1.24 | 0.87-1.77 | | 0.242 | 1.29 | 0.83-2.00 | 0.259 |
| CRAFITY |  |  | |  |  |  |  |
| 0 | Reference | | | | | | |
| 1 | 1.21 | 0.84-1.72 | | 0.306 | 1.46 | 0.92-2.30 | 0.107 |
| 2 | 1.93 | 1.36-2.75 | | <0.001 | 2.28 | 1.45-3.59 | <0.001 |
| NLR | 1.40 | 1.08-1.82 | | 0.0122 | 1.63 | 1.19-2.24 | <0.01 |
| PNI(<45/≥45) | 0.66 | 0.51-0.84 | | <0.001 | 0.64 | 0.47-0.86 | 0.00277 |
| PLR（0/1） | 1.36 | 1.1-1.69 | | <0.01 | 1.33 | 1.02-1.73 | 0.0383 |
| SII（0/1） | 1.90 | 1.34-2.68 | | <0.001 | 2.22 | 1.41-3.48 | <0.001 |
| LCR（0/1） | 0.50 | 0.36-0.71 | | <0.001 | 0.48 | 0.31-0.73 | <0.001 |
| CAR（0/1） | 1.54 | 1.24-1.92 | | <0.001 | 1.64 | 1.25-2.14 | <0.001 |
| mGPS |  |  | |  |  |  |  |
| 0 | Reference | | | | | | |
| 1 | 1.63 | 1.26-2.13 | | <0.001 | 1.77 | 1.27-2.46 | <0.001 |
| 2 | 2.01 | 1.30-3.11 | | <0.01 | 2.82 | 1.71-4.66 | <0.001 |

Abbreviations: α-FAtE: α-fetoprotein (**AF**), alkaline phosphatase (**A)** and eosinophil count(**E**); BCLC, Barcelona Clinic Liver Cancer; HBsAg, hepatitis B surface antigen; AST, aspartate aminotransferase; ALT, alanine aminotransferase; ALB, albumin; TBil, total bilirubin; WBC, leukocyte; CRP: C-reaction protein AFP, alpha-fetoprotein; CRAFITY C-reactive protein and alpha-fetoprotein in immunotherapy; PNI: prognostic nutritional index; NLR: neutrophil-to-lymphocyte ratio; PLR: platelet-to-lymphocyte ratio; SII: systemic immune-inflammation index; LCR: lymphocyte-to-CRP ratio; CAR: CRP-to-albumin ratio; mGPS: modified Glasgow prognostic score.

Supplementary table3: The results of Schoenfeld residuals analysis

| biomarker | Progression-free survival | Overall survival |
| --- | --- | --- |
|  | *P*-value | *P*-value |
| BCLC | **0.01** |  |
| Immunotherapy | **0.01** | 0.85 |
| Targeted therapy | **0.00** | 0.41 |
| α-FAtE | 0.41 | 0.18 |
| ALBI | 0.16 | 0.6 |
| Tumor number | 0.65 | 0.19 |
| Macrovascular invasion |  | 0.4 |
| Extrahepatic metastasis |  | **0.024** |
| CRAFITY | 0.41 | 0.57 |
| PNI | 0.35 | 0.36 |
| SII | 0.70 | 0.23 |
| CAR | 0.25 | 0.27 |
| mGPS | 0.61 | 0.2 |
| NLR | 0.58 | 0.21 |
| PLR | 0.47 | 0.057 |
| LCR | 0.24 | **0.04** |
| GLOBAL | 0.00 | 0.172 |

Abbreviations: α-FAtE: α-fetoprotein (**AF**), alkaline phosphatase (**A)** and eosinophil count(**E**); BCLC, Barcelona Clinic Liver Cancer; CRAFITY C-reactive protein and alpha-fetoprotein in immunotherapy; PNI: prognostic nutritional index; NLR: neutrophil-to-lymphocyte ratio; PLR: platelet-to-lymphocyte ratio; SII: systemic immune-inflammation index; CAR: CRP-to-albumin ratio; mGPS: modified Glasgow prognostic score.

Supplementary table4: The Concordance Index and time-dependent ROC of α-FAtE and other blood biomarkers

|  | Overall survival | | | | | Progression-free survival | | | | |
| --- | --- | --- | --- | --- | --- | --- | --- | --- | --- | --- |
| Peripheral blood biomarker | C-index | 12-months | 18-months | 24-months | 36-months | C-index | 12-months | 18-months | 24-months | 36-months |
| α-FAtE | 0.596 | 0.606 | 0.646 | 0.656 | 0.647 | 0.575 | 0.6 | 0.603 | 0.633 | 0.663 |
| CRAFIY | 0.581 | 0.616 | 0.6 | 0.613 | 0.637 | 0.574 | 0.603 | 0.557 | 0.603 | 0.636 |
| PNI | 0.547 | 0.564 | 0.563 | 0.559 | 0.563 | 0.532 | 0.543 | 0.573 | 0.575 | 0.573 |
| NLR | 0.545 | 0.574 | 0.537 | 0.542 | 0.565 | 0.525 | 0.526 | 0.53 | 0.546 | 0.585 |
| PLR | 0.549 | 0.595 | 0.54 | 0.549 | 0.574 | 0.543 | 0.58 | 0.551 | 0.555 | 0.588 |
| SII | 0.552 | 0.576 | 0.562 | 0.566 | 0.6 | 0.542 | 0.566 | 0.543 | 0.56 | 0.599 |
| LCR | 0.554 | 0.583 | 0.57 | 0.581 | 0.575 | 0.547 | 0.583 | 0.557 | 0.576 | 0.595 |
| CAR | 0.568 | 0.614 | 0.586 | 0.588 | 0.645 | 0.558 | 0.585 | 0.582 | 0.603 | 0.626 |
| mGPS | 0.584 | 0.615 | 0.606 | 0.613 | 0.616 | 0.555 | 0.597 | 0.58 | 0.608 | 0.616 |

Abbreviations: α-FAtE: α-fetoprotein (**AF**), alkaline phosphatase (**A)** and eosinophil count(**E**); CRAFITY C-reactive protein and alpha-fetoprotein in immunotherapy; PNI: prognostic nutritional index; NLR: neutrophil-to-lymphocyte ratio; PLR: platelet-to-lymphocyte ratio; SII: systemic immune-inflammation index; LCR: lymphocyte-to-CRP ratio; CAR: CRP-to-albumin ratio; mGPS: modified Glasgow prognostic score.
